# Dark-adapted red flash ERGs in healthy adults

**DOI:** 10.1007/s10633-018-9642-1

**Published:** 2018-06-01

**Authors:** R. Hamilton, K. Graham

**Affiliations:** 1 Department of Clinical Physics and Bio-Engineering, NHS Greater Glasgow and Clyde, Glasgow, UK; 2 College of Medical, Veterinary and Life Sciences, University of Glasgow, Glasgow, UK

**Keywords:** ISCEV standard, Dark-adapted red flash ERG, x-wave, Dark-adapted

## Abstract

**Purpose:**

The x-wave of the dark-adapted (DA) ERG to a red flash reflects DA cone function. This exploratory study of healthy adults aimed to investigate changes in the DA red ERG with flash strength and during dark adaptation to optimise visualisation and therefore quantification of the x-wave.

**Methods:**

The effect of altering red flash strength was investigated in four subjects by recording ERGs after 20 min dark adaptation to red flashes (0.2–2.0 cd s m^−2^) using skin electrodes and natural pupils. The effect of dark adaptation duration was investigated in 16 subjects during 20 min in the dark, by recording DA 1.5 red ERGs at 1, 2, 3, 4, 5, 10, 15 and 20 min.

**Results:**

For a dark adaption period of 20 min, the x-wave was more clearly visualised to weaker (< 0.6 cd s m^−2^) red flash strengths: to stronger flashes it became obscured by the b-wave. For red flashes of 1.5 cd s m^−2^, the x-wave was most prominent in ERGs recorded after 1–5 min of dark adaptation: with longer dark adaptation, it was subsumed into the b-wave’s rising edge.

**Conclusions:**

This small study suggests that x-wave visibility in healthy subjects after 20 min dark adaptation is improved by using flashes weaker than around 0.6 cd s m^−2^; for flash strengths of 1.5 cd s m^−2^, x-wave visibility is enhanced by recording after only around 5 min of dark adaptation. No evidence was found that interim red flash ERGs affect the dark-adapted state of the normal retina.

## Introduction

The dark-adapted (DA) ERG to a red flash has an initial positive peak called the x-wave [1, 2] which is seen only in species with cone-rich retinae [3]. X-wave amplitude is largest to wavelengths around 630 nm [2–4], and increases during dark adaptation, peaking within a few minutes [4–6]. Its peak time, unlike the b-wave, changes little with wavelength [7]. Visibility of the x-wave can be enhanced by using a dim background to suppress rods [4, 8].

No x-wave is evident in protanomalous subjects [4, 7, 9] nor in subjects with achromatopsia [7, 9]. Conversely, the x-wave is preserved but the b-wave is attenuated or absent in RDH5 retinopathy (fundus albipunctatus) [10, 11] and in vitamin A deficiency [12]. The DA ERG to a red flash is present—albeit with impaired kinetics [13]—in bradyopsia (RGS9/R9AP mutation), indicating preserved cone function, whereas the light-adapted white flash ERG is absent, a combination reported to be pathognomonic for the condition [14]. The x-wave is, therefore, interpreted as a measure of dark-adapted cone function. Although a DA red ERG is not part of the ISCEV ERG standard [15], it is used ‘sometimes’ or ‘often’ in around half of visual electrophysiology clinics [16]. Red flash strengths of 0.05–2.5 cd s m^−2^ [8, 10, 17–21] have been used, but some studies report clinical use of the red flash ERG without giving flash strength or spectral characteristics, instead describing flash strength “such that in a normal subject the amplitude of the rod component to the red flash is equivalent to that of the rod-specific response to a dim white flash (dark-adapted 0.01 cd s m^−2^)” [11, 12, 14, 22].

The x-wave can be swamped by the later, larger rod b-wave, appearing as only a shoulder which hampers quantification [20, 21]. Clearer visualisation and hence quantification of the x-wave might be achieved by a suitable combination of red flash strength and dark adaptation duration. This exploratory study aimed to investigate changes in the DA red ERG with flash strength and during dark adaptation.

## Methods

The study was approved by the Ethics Committee of the College of Medical, Veterinary and Life Sciences, University of Glasgow. Subjects gave informed, written consent.

### Subjects

Sixteen adult subjects (20–58 years old) without self-reported neurological or ocular conditions were recruited without incentive. Inclusion criteria were refractive errors of < 3 dioptres, and a normal Ishihara colour test result. The sample size was selected to power a concomitant study of shorter dark adaptation for ISCEV standard DA ERGs [23].

### Study design

Subjects were restricted to interior lighting for at least 1 h prior to any recordings. The test room was artificially lit. The first investigation explored the effect of altering red flash strength on four subjects (all female, aged 22–53). After 20 min dark adaptation, ERGs were recorded as described below from one random eye to red flashes of 0.2 or 0.3–2.0 phot cd s m^−2^ in 0.1 steps. A white 0.01 phot cd s m^−2^ ERG (DA 0.01 ERG) was also recorded at each step for comparison and to check for any evidence, e.g. reducing amplitude, that the retina was becoming light adapted.

**Fig. 1:**
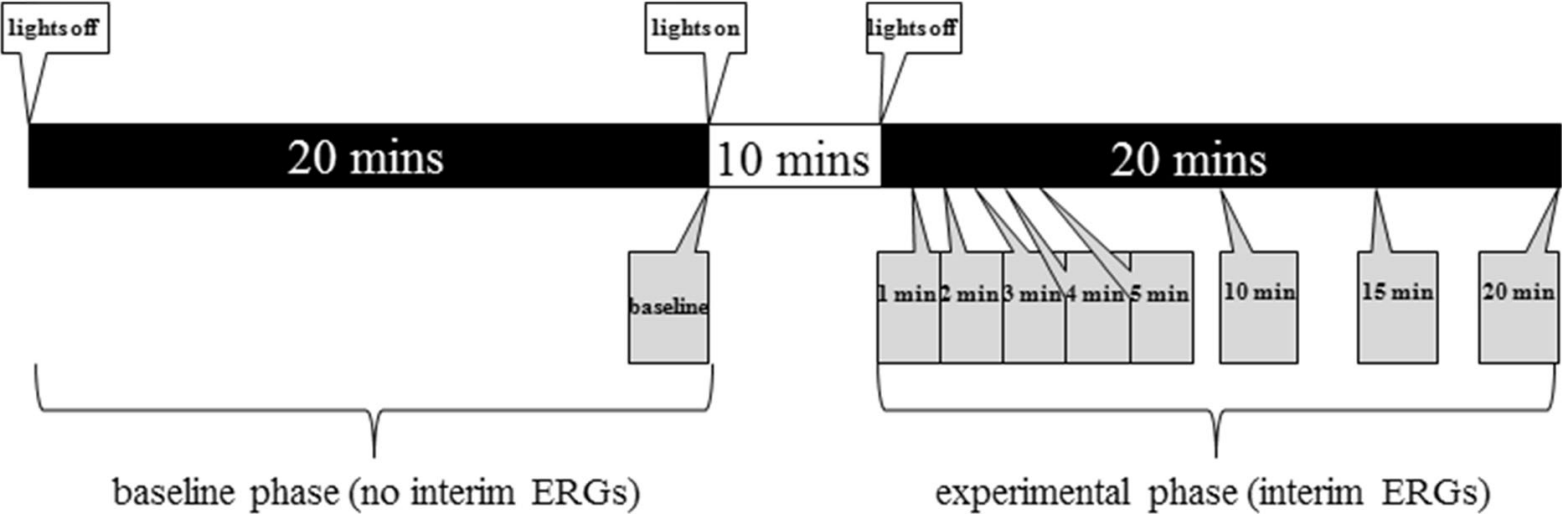
Timeline illustration of recording protocol. Black and white bands indicate dark and light adaptation, respectively. Boxes below the timeline indicate ERG recordings, labelled by duration of dark adaptation after which they were made

The second investigation explored the effect of altering duration of dark adaptation on the red flash ERGs, and was conducted as part of the concomitant study [23]. In the baseline phase of the experiment (Fig. 1), subjects were dark adapted for 20 min, at the end of which a DA 1.5 cd s m^−2^ red flash ERG (followed by ISCEV standard DA 0.01 and DA 3.0 ERGs) was recorded. Subjects were then light adapted to the artificial room lighting for 10 min before beginning the experimental phase of the protocol. In the experimental phase, dark adaptation was recommenced and interim DA 1.5 red ERGs were recorded during this second 20-min dark adaptation period at 1, 2, 3, 4, 5, 10, 15 and 20 min. The baseline phase was included in order to compare ERGs recorded after 20 min uninterrupted dark adaptation with those recorded after 20 min dark adaptation punctuated with multiple, interim ERGs, in order to test whether the experimental design itself affected the ERGs.

### ERGs

ERGs were recorded from both eyes using adhesive, disposable skin electrodes placed on the lower lid, referenced to skin electrodes at the ipsilateral temporal orbital rim. A ground electrode was placed on a mastoid. Skin was prepared to ensure low (< 5 kΩ) and matched impedances; amplifier bandpass was 0.3–300 Hz (IIR digital, 2 pole Bessel emulations), with a sampling frequency of 1000 Hz. In the interests of investigating protocols with greater patient test acceptability, no dilating drops were used, contrary to the stipulation of the ISCEV standard [15]. Pupil sizes were measured towards the end of the baseline period of dark adaptation using a half-moon rule with 0.5 mm precision and an infrared camera. Diameters ranged from 7 to 10 mm (median 8.5 mm), very similar to those from an earlier study on similar subjects (7–9 mm) with mydriasis [24]. A dim red fixation mark aided eye stability during recordings.

**Fig. 2:**
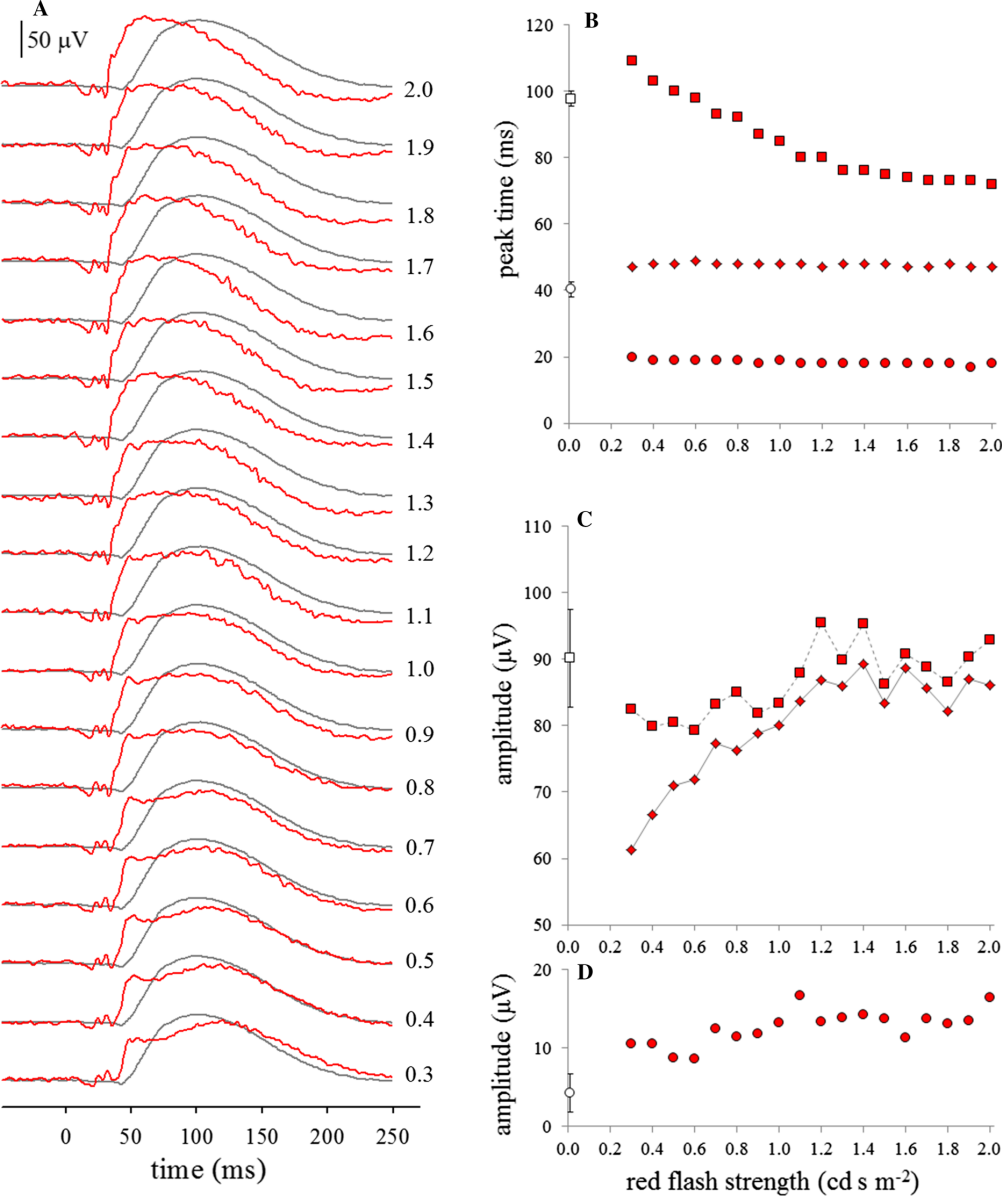
Illustrative red flash ERGs (red traces) and ISCEV standard DA 0.01 ERGs (grey traces) from subject #16, recorded after 20 min dark adaptation (**a**). The numbers to the right of each pair of traces indicate the strength of the red flash in photopic cd s m^−2^. Right panels show effect of red flash strength on subject #16’s peak times (**b**); x and b-wave amplitudes (**c**) and a-wave amplitudes (**d**). Circles: a-waves, diamonds: x-waves, squares: b-waves. Open symbols close to y-axes represent mean (sd) values for DA 0.01 ERGs for comparison

Stimulation and acquisition were driven by a visual electrophysiology system (Espion, Diagnosys LLC, Lowell, MA, USA). Ten ERGs were averaged with an inter-stimulus interval of 1 s to ensure adequately high SNR since skin electrodes were used. Flashes were generated by LEDs within a ganzfeld (ColorDome, Diagnosys LLC, Lowell, MA, USA) with stated peak wavelength *λ* = 635 nm and CIE coordinates *x* = 0.702, *y* = 0.298. Annual manufacturer’s calibration before and after the investigation showed no changes; values were confirmed for white flashes using a photometer (ILT1700, International Light Technologies, MA, USA) in integrating mode. Values given here are manufacturer’s nominal values. For the second investigation, a red flash strength of 1.5 (photopic) cd s m^−2^ was chosen somewhat arbitrarily, as it fell within the range of flash strengths described elsewhere for use with a preceding 20 min period of dark adaptation (0.05–2.5 cd s m^−2^ [8, 10, 17–21]); additionally, it generated a b-wave of a similar amplitude to that produced by the DA 0.01 ERG white flashes (Fig. 2).

Amplitudes of a-waves were measured from baseline, and x- and b-waves were measured from a-wave troughs. No systematic inter-ocular ERG differences existed, so parameters from eyes of each subject were averaged [25]. Data were treated nonparametrically because of the small sample size and some skew.

## Results

### ERG changes with red flash strength

The red flash ERG grew in amplitude as flash strength increased for all four subjects tested (Fig. 2). The a-wave was present, typically at around 17–20 ms. Two further troughs were evident, typically shallower, at around 25–28 ms and at around 30–37 ms, depending on flash strength. The x-wave was also present, typically at around 45–50 ms, and was more clearly seen at lower than at higher flash strengths: as flash strength increased, it became larger, but was increasingly obscured by the b-wave, appearing as a shoulder on the b-wave rising edge. At lower flash strengths, the b-wave was also clearly visualised at around 100 ms, with a similar form to the b-wave of the dim white flash ERG. As red flash strength increased, the b-wave shortened and merged with the x-wave peak.

The white flash DA 0.01 ERGs recorded at each step showed no evidence of reducing in amplitude over the 10 ERGs used for the average in each step, nor over the whole investigation for any subject, despite a 1-s inter-stimulus interval, rather than the 2 s stipulated in the ERG standard [15]. Similarly, the red flash ERGs showed no evidence of reducing in amplitude over the 10 ERGs used for the average in each step, suggesting that, even for relatively strong red flashes, a 1-s inter-stimulus interval was adequate to maintain the dark-adapted state of the retina.

### ERG changes with duration of dark adaptation

**Fig. 3:**
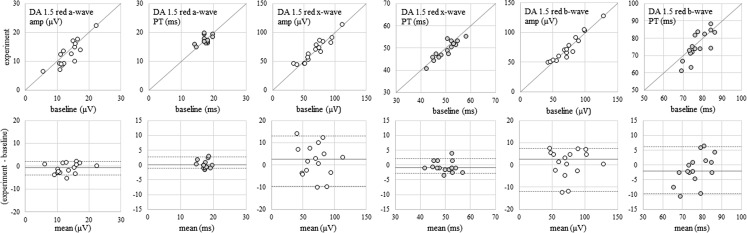
Upper panels: scatterplots of ERG parameters recorded at the end of baseline phase versus end of experimental phase for all 16 subjects. Grey diagonal lines indicate equality. Lower panels: corresponding difference plots. Grey horizontal lines indicate the median difference between experimental and baseline recordings, and grey dashed lines indicate the 5th and 95th percentiles of the difference

Comparing ERGs recorded at the end of the baseline phase with those recorded at the end of the experimental phase revealed no statistically significant differences in ERG parameters (Mann–Whitney *U* tests, *p* values all ≥ 0.50), establishing that interim ERGs recorded during the experimental phase did not affect the measured parameters of ERGs recorded at the end of the dark adaptation period (Fig. 3). This also implies that delivering red flashes with 1-s inter-stimulus intervals does not affect the adaptation state of the retina, notwithstanding the additional white flashes delivered during the experimental phase [23].

**Fig. 4:**
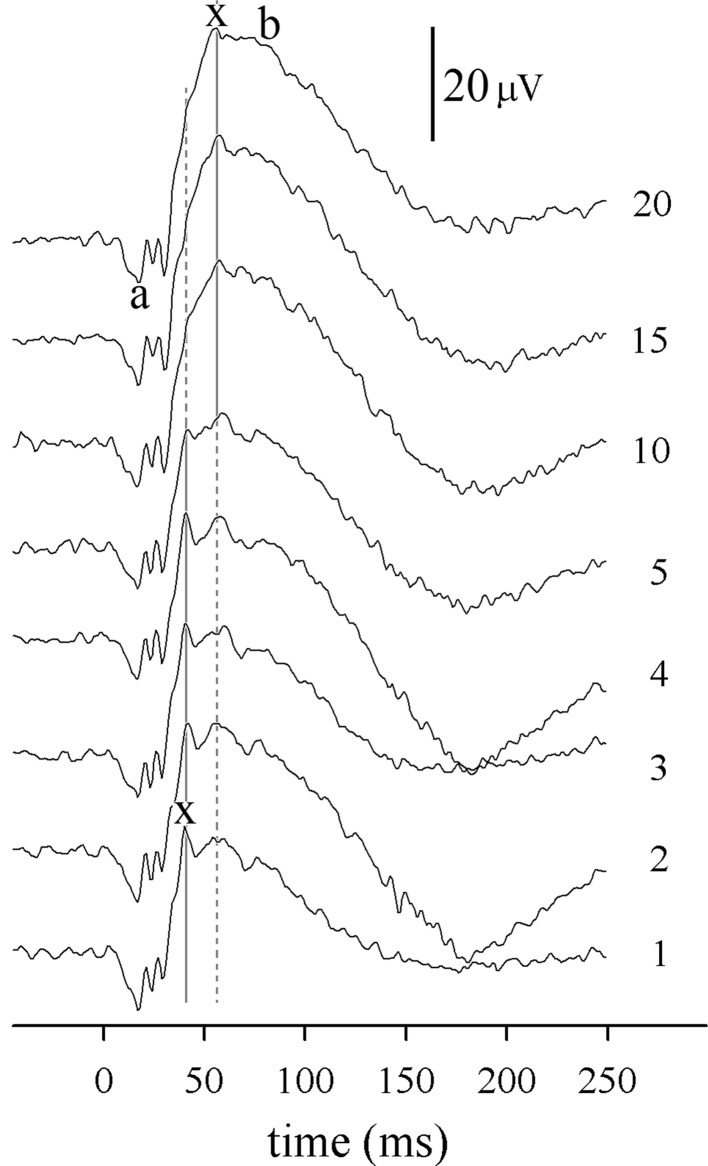
Illustrative DA 1.5 red ERGs from a typical subject (#8). The numbers to the right of each trace indicate the duration of dark adaptation (minutes) before each ERG. Vertical grey lines mark the two positive peaks which exchange dominance (largest amplitude) as dark adaptation proceeds. The largest peak is measured as the x-wave (continuous line). Note the triple trough which forms the a-wave, with troughs typically at around 17, 24 and 31 ms

The morphology of the DA 1.5 red ERG changes during dark adaptation (Fig. 4). A series of oscillations at the a-wave and rising edge of the b-wave are evident, with the largest positive peak—the x-wave—being most prominent at around 40 ms in ERGs recorded after 1–5 min of dark adaptation. As the presumably rod-driven, later b-wave gains amplitude during dark adaptation, this 40-ms x-wave peak becomes subsumed into the b-wave’s rising edge, often being no longer apparent, or present as only a shoulder with no following trough. A later oscillation, usually > 50 ms, is more clearly visualised as the x-wave than the 40-ms peak in ERGs recorded after 10–20 min of dark adaptation.

**Fig. 5:**
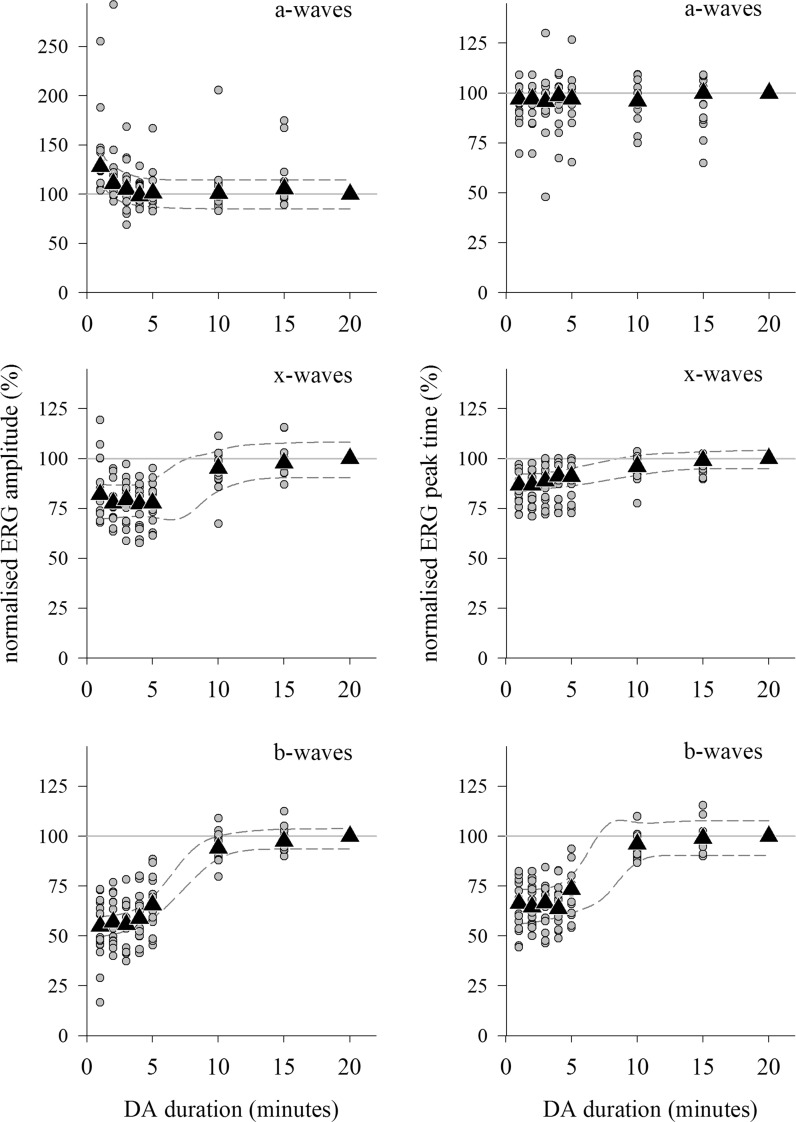
DA red 1.5 ERG changes during dark adaptation. Left: amplitudes. Right: peak times. Circles: individual subject’s normalised data points; triangles: median values; dashed lines: 95% prediction intervals of growth curves fitted to median data; solid horizontal line highlights the 100% level. Data are normalised relative to values after 20 min dark adaptation, hence the lack of variability at 20 min. Note change of scale for a-wave amplitudes

The development of the DA red 1.5 ERG during dark adaptation was quantified by normalising individual subject’s ERG parameters to those of their 20 min DA ERG (Fig. 5). The red a-wave amplitude reduced a little over the first 3 min of dark adaptation, while peak times did not change at all over the whole 20 min. The x-wave amplitude increased, and peak time lengthened between five and 10 min of dark adaptation, primarily due to the later peak at ~ 50 ms becoming dominant as the growing b-wave obscured the earlier x-wave peak at ~ 40 ms. The b-wave amplitude increased, and peak time shortened with lengthening dark adaptation as might be expected for a rod system b-wave.

Summarised reference data are presented in Table 1 for the DA 1.5 red flash ERG after 5, 10 and 20 min of dark adaptation: data are presented as ranges as the sample size is inadequate for percentiles with confidence intervals [26]. These data do not change substantially with duration of dark adaptation, except for the x-wave peak time which more tightly defined after 5 min in the dark than after longer dark adaptation.

**Table 1:** Summarised reference data (ranges) for DA 1.5 red flash ERGs, skin electrodes, undilated pupils, *N *= 16 subjects

Duration of dark adaptation (min)	a-wave		x-wave		b-wave	
	Amplitude (µV)	PT (ms)	Amplitude (µV)	PT (ms)	Amplitude (µV)	PT (ms)
5	5.2–24	15–20	31–100	41–50	21–97	62–89
10	6.3–25	15–19	38–110	41–58	40–128	66–92
20	6.6–22	15–20	45–114	41–56	50–129	62–89

*PT* peak time

## Discussion

In this small, exploratory study of healthy adult subjects, we found that after 20 min of dark adaptation, the x-wave was more clearly visualised with weaker (about 0.6 cd s m^−2^ or less) than with stronger flashes, as described elsewhere [18]. We also found that that with a relatively strong 1.5 cd s m^−2^ red flash, the x-wave was more clearly visualised after shorter (about 5 min or less) than longer dark adaptation, also confirming findings elsewhere [6].

The subjects in this study were mostly in their second or third decades, so findings cannot be generalised to all age groups. As an exploratory study, we chose a minimally invasive protocol with non-corneal electrodes and no mydriasis: this will have resulted in higher signal-to-noise ratios than usually found with corneal electrodes. This is unlikely to affect conclusions since mostly relative outcome measures were used, but signal noise increases uncertainty of peak labelling in some instances.

The DA red flash ERG is quite widely used, and the International Society for Clinical Electrophysiology of Vision has recently prepared a new Extended Protocol to inform current and potential users. Its utility lies in an extant x-wave revealing the presence of functioning, dark-adapted cones. Thus, the visibility of the x-wave is of primary importance for the optimisation of protocols. The current findings suggest that x-wave visibility in normal subjects is impaired by flashes stronger than around 0.6 cd s m^−2^ (for 20 min dark adaptation), or by dark adaptation longer than around 5 min (for flash strengths of 1.5 cd s m^−2^). Other combinations remain uninvestigated. It seems feasible that the ISCEV standard dark-adapted ERG protocol could incorporate a dim red flash ERG delivered at some point during the currently stipulated 20 min of dark adaptation, adding no further burden of time to the patient or tester. These data also suggest that additional flashes, even delivered once per second, are unlikely to affect the dark-adapted state of the normal retina, although this may not be the case for patients with retinal dysfunction.
